# Generation and characterization of individual-specific induced pluripotent cells from patient-derived lymphoblastoid cell lines

**DOI:** 10.1186/1750-1326-8-S1-P53

**Published:** 2013-09-13

**Authors:** Shadaan Zulfiqar, Radhika Menon, Tara Atmaram, Meera Purushottam, Matthew Varghese, Sanjeev Jain, Mitradas Panicker, Odity Mukherjee

**Affiliations:** 1 NCBS-TIFR, Bangalore, Karnataka-560065, lndia; 2 Department of Psychiatry, NIMHANS, Bangalore, Karnataka-560029, India

## Background

Alzheimer’s disease (AD) is a common neurodegenerative disorder that leads to cognitive impairment. In India, the prevalence of AD is currently ~3.5 million but due to its large population and the recent increase in longevity, is projected to increase to 7-9 million by 2020. Thus, AD is a significant burden of disease in our country. Genetic variations play a major role in AD. The apolipoprotein-E gene and in particular its ε4 allele account for 20-50% of the total genetic variance in disease risk. One copy of ε4 allele increases risk for AD by ~threefold, and two copies, by more than ten-fold. But still, ε4 allele is neither necessary nor sufficient for the development of the disease, indicating involvement of other genetic risk factors, alone or in conjunction with the ε4 allele underlying disease predisposition.

## Materials and methods

Patient derived Lymphoblastoid cells (LCLs) carrying pre-disposing APOE4 allele was used to generate induced pluripotent stem cells (iPSCs) using episomal plasmids. Post characterization, theiPSC lines were differentiated into neuronal lineage using retinoic acid induction method. Whole genome transcriptome analysis was performed using RNA isolated from MAP2/Tuj1 positive cells (17-22 DIV). Protein expression studies (by Western Blot) for key molecules was also performed to validate the above results.

## Results

We have successfully generated iPSC lines from AD patients and cognitively normal controls (n=6). These lines display human embryonic stem-cell like morphology and transcription profile. These cells gave rise to neurons on directed differentiation that were positive for neuronal markers MAP2 and Tuj1. Whole genome transcriptome analysis of differentiating neurons derived from patient iPSCs with varying APOE genotype showed significant differential expression of coding and pseudogenes in implicated pathways. Briefly, genes encoding for focal adhesion molecules, glutamatergic synapses, axon guidance and cell adhesion protein showed differential expression patterns. Preliminary protein expression studies performed on parent LCL lines have confirmed some of transcriptome signals suggesting usefulness of peripheral models to interrogate complex phenotypes like AD.

## Conclusion

Most patient-specific iPSC lines reported use viral transduction as the mode of gene delivery for reprogramming. This may affect the phenotype and mechanism of the disease in question. We have generated iPSC lines using an integration-free method using LCLs. the derived LCL lines are stable for a large number of passages and serve as an alternate cell model to test experimental leads. As a preliminary step to test the validity of patient derived iPSCs to model AD, we have used whole genome RNA sequencing analysis. We show APOE allele- specific difference in expression profile of genes in key disease pathways. We are currently validating these leads in a larger patient sample.

